# Supplementary material for: Comparative multiomics analysis of cell physiological state after culture in a basket bioreactor
Source: Sci Rep. 2022 Nov 23;12:20161. doi: 10.1038/s41598-022-24687-4 (PMC9686226; doi:10.1038/s41598-022-24687-4)

23666048.1:98288717:98288824:-@NW_023666048.1:98288717:98288894:-@NW_023666048.1:98289112:98289

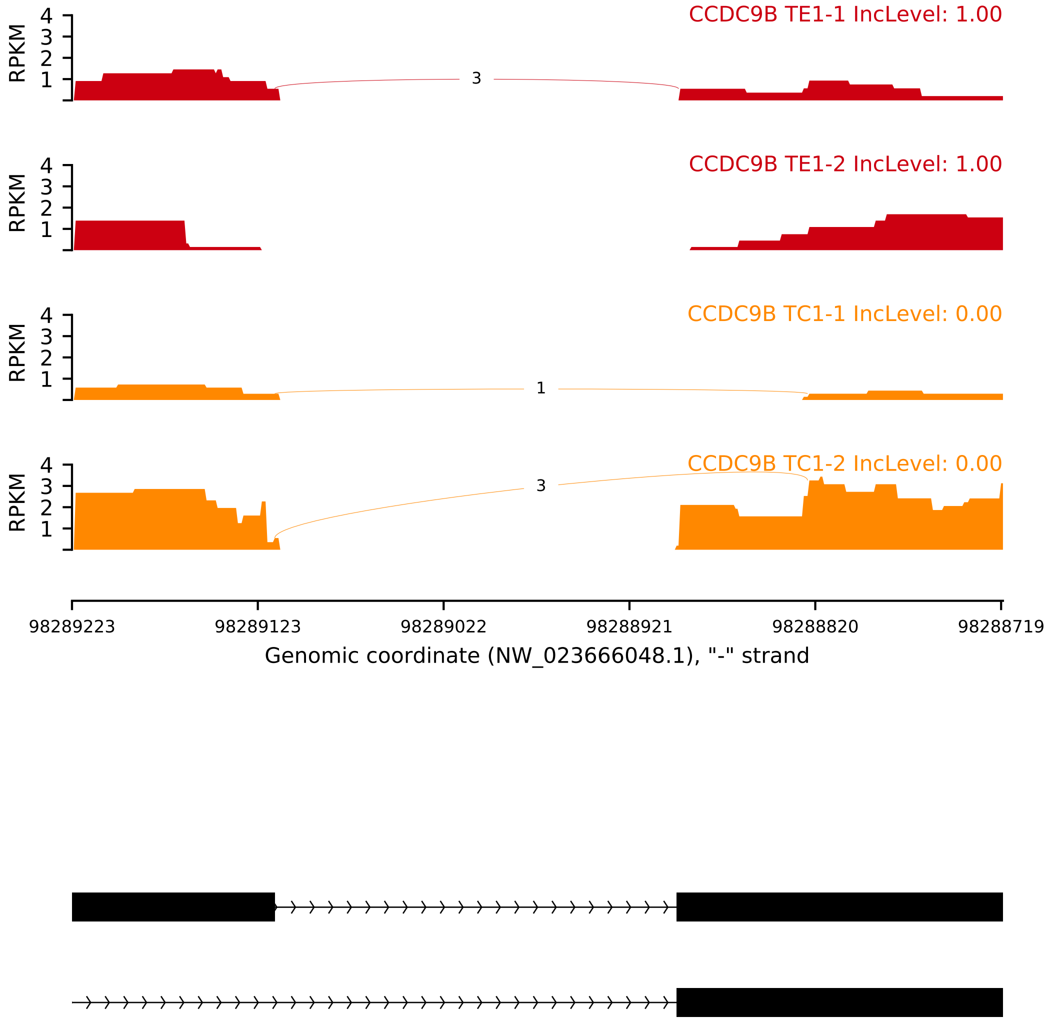

Supplement: Supplementary file 1 — Supplementary Information 1. [file 41598_2022_24687_MOESM1_ESM.zip › raw data/Transcriptomic raw data/7.AS/2.dasplot/TE1vsTC1/A3SS/5_CCDC9B_NW_023666048.1_98288717_98288824_-@NW_023666048.1_98288717_98288894_-@NW_023666048.1_98289112_98289222_-.pdf]
